# Lipopolysaccharide Cross-Tolerance Delays Platelet-Activating Factor-Induced Sudden Death in Swiss Albino Mice: Involvement of Cyclooxygenase in Cross-Tolerance

**DOI:** 10.1371/journal.pone.0153282

**Published:** 2016-04-11

**Authors:** Shancy Petsel Jacob, Chikkamenahalli Lakshminarayana Lakshmikanth, Vyala Hanumanthareddy Chaithra, Titus Ruth Shantha Kumari, Chu-Huang Chen, Thomas M. McIntyre, Gopal Kedihitlu Marathe

**Affiliations:** 1 Department of Studies in Biochemistry, University of Mysore, Manasagangothri, Mysuru, 570006, Karnataka, India; 2 Department of Zoology, St. Philomena’s College, Bannimantap, Mysuru, 570015, Karnataka, India; 3 Vascular and Medicinal Research, Texas Heart Institute, Houston, Texas, 77225–0345, United States of America; 4 Department of Cellular and Molecular Medicine (NC10), Cleveland Clinic Lerner Research Institute, 9500 Euclid Avenue, Cleveland, Ohio, 44195, United States of America; University of Leuven, Rega Institute, BELGIUM

## Abstract

Lipopolysaccharide (LPS) signaling through Toll-like receptor-4 (TLR-4) has been implicated in the pathogenesis of many infectious diseases. Some believe that TLR-mediated pathogenicity is due, in part, to the lipid pro-inflammatory mediator platelet-activating factor (PAF), but this has been questioned. To test the direct contribution of PAF in endotoxemia in murine models, we injected PAF intraperitoneally into Swiss albino mice in the presence and absence of LPS. PAF alone (5 μg/mouse) caused death within 15–20 min, but this could be prevented by pretreating mice with PAF-receptor (PAF-R) antagonists or PAF-acetylhydrolase (PAF-AH). A low dose of LPS (5 mg/kg body wt) did not impair PAF-induced death, whereas higher doses (10 or 20 mg/kg body wt) delayed death, probably via LPS cross-tolerance. Cross-tolerance occurred only when PAF was injected simultaneously with LPS or within 30 min of LPS injection. Tolerance does not appear to be due to an abundant soluble mediator. Histologic examination of lungs and liver and measurement of circulating TNF-α and IL-10 levels suggested that the inflammatory response is not diminished during cross-tolerance. Interestingly, aspirin, a non-specific cyclooxygenase (COX) inhibitor, partially blocked PAF-induced sudden death, whereas NS-398, a specific COX-2 inhibitor, completely protected mice from the lethal effects of PAF. Both COX inhibitors (at 20 mg/kg body wt) independently amplified the cross-tolerance exerted by higher dose of LPS, suggesting that COX-derived eicosanoids may be involved in these events. Thus, PAF does not seem to have a protective role in endotoxemia, but its effects are delayed by LPS in a COX-sensitive way. These findings are likely to shed light on basic aspects of the endotoxin cross-tolerance occurring in many disease conditions and may offer new opportunities for clinical intervention.

## Introduction

Microbial products induce a shift in the innate immune system towards a pro-inflammatory phenotype by activating a family of pattern-recognizing receptors popularly known as Toll-like receptors [1].The downstream signaling events of these receptors are critical in the pathogenesis of many infectious disease complications, such as endotoxemia/sepsis [2]. Despite the substantial improvement in critical care, sepsis still accounts for many deaths in intensive care units globally [3]. A pleiotropic mediator often implicated in sepsis is the bacterial endotoxin lipopolysaccharide (LPS) [4–5]. LPS interacts with the Toll-like receptor-4 (TLR-4), along with other accessory components, to generate a battery of pro-inflammatory cytokines and lipid mediators that promote a systemic inflammatory response–the hallmark of sepsis [6]. Although LPS is a widely studied microbial product that has been targeted for the treatment of sepsis, not a single drug has been found to successfully treat sepsis in more than 100 clinical trials conducted so far [7]. Therefore, a better understanding is needed of sepsis in general and the role of TLR-4 agonists in this process before new therapeutics against sepsis is developed.

Some of the complications associated with the activation of TLR-4 are attributed to the endogenously generated phospholipid mediator platelet-activating factor (PAF) [8–9]. PAF is chemically identified as 1-alkyl-2-acetyl*sn*-3 glycerophosphocholine [10–14] and was originally described as a fluid-phase lipid mediator capable of inducing anaphylactic shock [15]. Many structural analogs of PAF, collectively referred to as “PAF-mimetics” or “PAF-like lipids,” that arise from the oxidative fragmentation of intact phospholipids have also been documented to be involved in many pathological conditions, including sepsis [16–19]. Both PAF and PAF-like lipids are recognized by the PAF receptor (PAF-R), a typical G protein-coupled receptor predominantly found on the surface of cells of the innate immune system [20–21], however, a separate receptor for related PAF-like lipids has also been suggested [22]. Inactivation of PAF and PAF-like lipids is catalyzed by the removal of *sn*-2 residues by a family of related enzymes called PAF-acetylhydrolase (PAF-AH) [23–26]. The plasma form of PAF-AH has been thoroughly characterized. Experimental and clinical observations suggest that PAF signaling plays a critical role in sepsis and related disorders [27–28]. For example, blocking PAF-R activation using specific PAF-R antagonists protects animals from symptoms of endotoxemia [29–33]. In addition, a high level of PAF was found in bronchoalveolar lavage fluid from people with acute respiratory distress syndrome (ARDS), a disorder associated with sepsis [34]. A role for PAF in ARDS has been supported by the results of experiments using transgenic mice that over-expressed PAF-R [35]. Serum levels of PAF were also found to be increased in experimental animals with endotoxemia, but these animals were protected by using PAF-R antagonists [36]. Graham et al [37] observed a 50% decrease in plasma PAF-AH activity in patients with sepsis and showed that the half-life of PAF in the plasma was prolonged in patients with the worst outcomes. Overexpression of the PAF-R makes mice hypersensitive not only to PAF but also to endotoxin [38–39]. Conversely, deleting the PAF-R makes mice less susceptible to anaphylaxis and inflammation in the lungs [40], a tissue often affected during endotoxemia [41–46]. Moreover, administration of exogenous PAF-AH protects animals from undergoing anaphylactic shock [47] and lung inflammation [48]. For instance, studies by Teixeira-da-Cunha et al [49] have shown that bacterial clearance is increased in septic mice treated with rPAF-AH. Studies by Watanabe et al [9] showed that neutrophils challenged with endotoxin produced PAF once they had adhered to a surface; a significant portion of this PAF was released in association with microparticles.

Thus, activated neutrophils may propagate and amplify the response to endotoxin via PAF. Contrary to these findings, a few studies have suggested a protective role for PAF and PAF-like lipids in models of endotoxemia [50–53], challenging the beliefs that PAF is pro-inflammatory in nature and that PAF-AH has anti-inflammatory effects.

To resolve this controversy and better understand the role of PAF in endotoxemia, we injected Swiss albino mice with PAF alone or in combination with the TLR-4 agonist LPS. PAF alone (5 μg/mouse) caused sudden death in these animals, but hyperactivating the TLR-4 with LPS delayed this event, probably by inducing endotoxin cross-tolerance, a phenomenon that is not clearly understood but has been reported to occur in several studies [54–63]. Endotoxin tolerance is generally defined as a situation in which previous exposure to low doses of LPS confers protection against the potentially detrimental consequences of a subsequent higher dose of LPS (homologous ligand) in animals or isolated cells. This tolerance induced by LPS pre-exposure does not only occur upon subsequent challenge with LPS, but also upon challenge with unrelated (heterologous) ligands, which is termed LPS/endotoxin cross-tolerance [59]. Although cross-tolerance has been previously reported, the underlying mechanism is still unclear. In this study, we show that hyperactivation of TLR-4 causes temporal cross-tolerance to the lethal effects of PAF in Swiss albino mice.

To understand the molecular mechanism underlying LPS cross-tolerance, we pretreated animals with both aspirin (a non-specific COX inhibitor [64]) and NS-398 (a specific COX-2 inhibitor [65]). Aspirin (20 mg/kg body wt) partially abolished PAF-induced sudden death and amplified the effects of cross-tolerance due to TLR-4 hyperactivation on PAF-induced sudden death. In contrast, NS-398 (20 mg/kg body wt) conferred complete protection against the lethal effects of PAF and also enhanced the cross-tolerance exerted by LPS. These studies confirm the pro-inflammatory nature of PAF and reveal an as yet unrecognized role for the COX pathway in LPS cross-tolerance in Swiss albino mice. Understanding how the COX pathway is involved in endotoxin cross-tolerance may provide new perspectives on the treatment of sepsis and other inflammatory disorders involving multiple mediators.

## Materials and Methods

LPS from *Escherichia coli* O111:B4 and aspirin were purchased from Sigma Chemicals Co. (St. Louis, MO). BN-52021, a Ginkgolide PAF-R antagonist, was purchased from BIOMOL Research laboratories (Plymouth Meeting, PA). PAF (C16), lysoPAF, C4 PAF, and lysoPC were obtained from Avanti Polar Lipids (Alabaster, AL). NS-398 was purchased from Cayman Chemical (Ann Arbor, MI). *o*-dianisidine and hydrogen peroxide (H_2_O_2_) were procured from Sisco Research Laboratories Pvt. Ltd. (Mumbai, India). Human serum albumin was obtained from Talecris Biotherapeutics Inc. (Duhram, NC). rPAF-AH was purchased from ICOS Corp. (Bothell, WA). Eosin was obtained from SD Fine Chemicals (Mumbai, India), and hematoxylin was obtained from Merck (Whitehouse Station, NJ). TNF-α and IL-10 ELISA kits were purchased from USCN Life Sciences Inc. (Buckingham, UK). WEB-2086, a thieno-triazolodiazepine PAF-R antagonist, was a generous gift from Boehringer Ingelheim Pharmaceuticals (Ridgefield, CT).

### Animals

Approval for our animal experiments was obtained from the Institutional Animal Ethical Committee (IAEC) of the University of Mysore, India (No. UOM/IAEC/14/2013). Swiss albino, C57BL/6J, and BALB/c mice (age, 8–10 weeks; weight, 20-25g) were used in the study. The Swiss albino mice were obtained from Central Animal Facility, University of Mysore, India, and the C57BL/6J and BALB/c mice were obtained from the National Centre for Laboratory Animal Sciences, National Institute of Nutrition, Hyderabad, India. All animals were housed with adequate ventilation, food, and water (available *ad libitum*). The mortality aspects of all the experimental protocols were specifically reviewed and approved by the IAEC of the University of Mysore, India and were in accordance with the guidelines provided by the Committee for the Purpose of Control and Supervision of Experiments on Animals (CPCSEA). To determine the effects of PAF and LPS on the survival of Swiss albino mice, we administered PAF, LPS, or a combination of both in varying concentrations and monitored the animals for survival for the required time interval. During the study, the animals were visually monitored at regular intervals (every 15 min) for dehydration (as seen by loss of skin elasticity), hunched posture, increased or labored respiration, failure to explore cage when disturbed, and deteriorating body condition. Animals injected with PAF alone (5 μg/mouse) or in conjunction with LPS (5 mg/kg body wt) died in 15–20 min, probably due to enhanced coagulation of blood induced by PAF. We were unable to use an alternative humane endpoint for these experiments because death occurred so rapidly that we were not confident enough in the outcome to interfere by using pain killers or other medications or by terminating the experiment before we were sure about the lethal outcome. However, there were no unintended deaths of the animals during the study. Based on recommendations from the Institutional Animal Ethical Committee (IAEC) and the animal caretaker at the Central Animal Facility, University of Mysore, India, the animals that survived to the end of the experiments (24 h or 6 days, according to experimental requirements) were euthanized by administering pentobarbital sodium salt intraperitoneally (30 mg/kg body wt), and their organs were used for histological examination.

### Effect of intraperitoneal injection of PAF in mice

To determine the effect of PAF on the survival of Swiss albino mice, we divided the animals into 6 groups, each containing 6 animals. A stock solution of PAF was made in methanol, and the required aliquot was dried under a stream of nitrogen. PAF was then reconstituted in PBS containing 0.1% human serum albumin and sonicated (Vibracell, Sonic Instrument Inc, NJ, USA) before use. The bioactivity of PAF was routinely checked before the experiments by assessing platelet aggregation in platelet-rich plasma. Testing with 80nM PAF consistently showed >80% platelet aggregation (although the sensitivity of platelets varied from donor to donor for unknown reasons). Generally, a standard dose of 5 μg of PAF per mouse was used for all experiments, except for the dose-response studies. Increasing doses of PAF (0–5 μg) were made in a total volume of 0.5 mL PBS containing 0.1% albumin and were administered intraperitoneally into Swiss albino mice. In some experiments, PAF (5 μg/mouse) was also intraperitoneally administered into C57BL/6J and BALB/c mice. Working PAF solutions were not stored for more than 24 h. Experiments were also performed to test the effects of lysoPAF and lysoPC at two doses: 5 and 50 μg per mouse. After each treatment, animals were monitored for up to 6 days for survival.

### Effect of rPAF-AH and PAF-R antagonists on PAF-challenged mice

Stock solutions of WEB-2086 and BN-52021 were made in DMSO, and appropriate aliquots (20 mg/kg body wt) were injected into animals 30 min before PAF (5 μg/mouse) was injected. In animal experiments involving pretreatment with PAF-AH (25 μg/mouse), animals were given a recombinant form (rPAF-AH) of the enzyme intraperitoneally 30 min before PAF was injected. Control animals received the vehicle used for rPAF-AH formulation (sodium citrate, sucrose, pluronic, and Tween-80). Survival time was monitored for up to 6 days.

### Effect of TLR-4 agonist on PAF-induced death in Swiss albino mice

To determine the effect of TLR-4 agonist on PAF-induced death, animals were intraperitoneally injected with PAF (5 μg/mouse) immediately after receiving either a low dose (5 mg/kg body wt) or high dose (10 or 20 mg/kg body wt) of LPS. In LPS cross-tolerance studies, PAF (5 μg/mouse) was co-administered intraperitoneally with LPS or administered at designated time intervals. To test the possibility that a soluble mediator may delay or provide protection against PAF-induced death during LPS cross-tolerance, we collected 100 μL of serum from mice injected with LPS (20 mg/kg) + PAF (5 μg/mouse) 1 h after treatment and intraperitoneally injected it into naive animals 30 min before injecting them with PAF (5 μg/mouse). All the animals were monitored for up to 6 days for survival. Death time was noted, and the tissues were removed and fixed as described below.

### Effect of COX inhibitors on PAF-induced death and LPS cross- tolerance

Next, to determine whether COX inhibitors have any effect on PAF-induced sudden death, we treated the animals with aspirin (10 or 20 mg/kg body wt) or NS-398 (20 mg/kg body wt) 30 min before administering a lethal dose of PAF (5 μg/mouse). To understand the mechanism of cross-tolerance occurring during hyperactivation of TLR-4, we intraperitoneally injected the animals with aspirin (10 or 20 mg/kg body wt) or NS-398 (20 mg/kg body wt) 30 min before injecting them with LPS (20 mg/kg) + PAF (5 μg/mouse). The animals were monitored for up to 6 days for survival.

### Histological Analysis

The lungs and livers excised from the euthanized animals were fixed for 24 h in Bouin’s fixative (picric acid: formaldehyde: glacial acetic acid, 30:10:2 v/v), dehydrated with increasing concentrations of ethanol, and embedded in paraffin. Tissues were cut into 5-micron–thick sections using a microtome (R. Jung AG, Germany) and stained with hematoxylin and eosin.

### Quantification of tissue myeloperoxidase (MPO)

The extent of leukocyte infiltration into the organs was indirectly estimated by quantifying tissue MPO levels using the method reported by Bradley et al [66]. Briefly, the tissues were homogenized in 50mM sodium phosphate buffer (pH 6.0) containing 0.5% hexadecyltrimethylammonium bromide (HTAB) and sonicated after repeated freeze-thaw cycles. The homogenates were centrifuged at 40,000 x g for 15 min at 4°C, and the protein in the supernatant was quantified using the Lowry method [67]. To measure MPO activity, 100 μL aliquots of the supernatants were mixed with 2.9 mL of 50mM sodium phosphate buffer (pH 6.0) containing 167 μg/mL of *o*-dianisidine and 0.0005% H_2_O_2_. The change in absorbance at 460nm was recorded with a UV-Visible spectrophotometer (Biomate 3S, Thermo Scientific, USA), and MPO activity was expressed in units (one unit of MPO activity = one micromole of peroxide degraded per milligram of protein per minute at 25°C).

### Measurement of the levels of circulating TNF-α and IL-10

Levels of circulating TNF-α and IL-10were measured in serum samples by using ELISA kits, as per the manufacturer’s instructions. The mice were anesthetized and whole blood was collected from the carotid artery using a 1 mL syringe. The serum was then separated from the whole blood. Generally, blood was collected 4 h after injection, unless stated otherwise. Animals injected with PAF alone (5 μg/mouse) or with 5 mg/kg LPS + PAF (5 μg/mouse) died in 15–20 min. Therefore, blood was collected from these animals 7 min after injection.

### Statistical analysis

Results for the animal experiments are representative of atleast 3 independent experiments. Statistical significance among groups was determined by one-way analysis of variance (ANOVA). The values are presented as mean ± SD.

## Results

### Intraperitoneal injection of PAF causes sudden death in Swiss albino mice within 15–20 min

**Fig 1:**
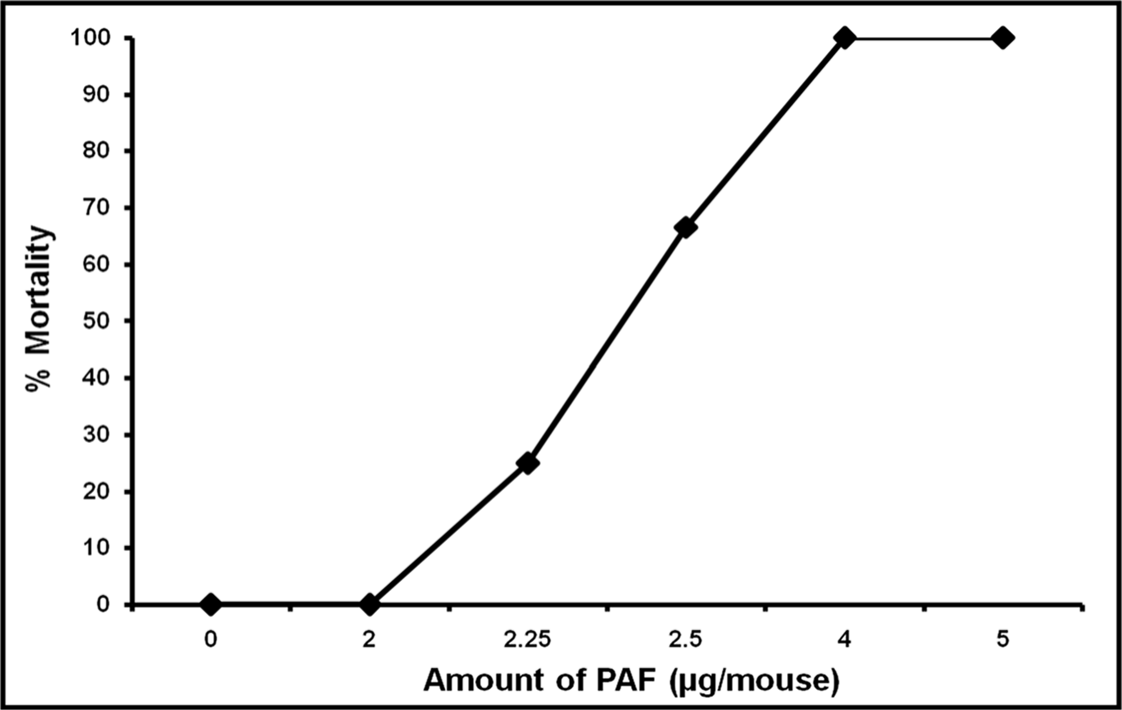
Dose-dependent effect of PAF on mortality in Swiss albino mice. The indicated amounts of PAF were aliquoted from a stock of 5 mg/mL in methanol, evaporated under a stream of nitrogen, reconstituted in phosphate buffered saline (PBS) containing 0.1% albumin, and sonicated. The animals were divided into 6 groups, each containing 6 animals. Each mouse was intraperitoneally injected with the designated dose of PAF in a total volume of 0.5 mL of PBS containing 0.1% albumin. Control animals received 0.5 mL PBS containing 0.1% albumin. The animals were monitored for survival for up to 6 days. The results are representative of 3 independent trials.

A variety of studies have shown that PAF has pro-inflammatory effects [10–11, 13, 29, 68–72]. However, a few reports have claimed that PAF and PAF-like lipids play a protective role in LPS-mediated endotoxemia [50–53]. Determining the cause of this discrepancy is critical for designing therapeutics to treat sepsis and for understanding the basic biology of PAF (reviewed in 18). To resolve the issue, we intraperitoneally injected Swiss albino mice with increasing amounts of PAF (0–5 μg/mouse) (Fig 1). We found that 50% of the mice died when given a 2.30 μg dose of PAF; therefore, we routinely used 5 μg as a lethal dose. At this dose, mice died within 15–20 min of injection in all the experiments performed. We also noticed that drawing blood from these mice was difficult due to enhanced coagulation of the blood. In contrast, the same dose of PAF (5 μg/mouse) was not lethal to C57BL/6J and BALB/c mice (Table 1). The specificity of PAF-induced death in Swiss albino mice was further confirmed with 2 sets of experiments: (1) by pre-injecting the mice with 2 structurally unrelated, yet specific, PAF-R antagonists, namely BN-52021 and WEB-2086 (20 mg/kg body wt) and (2) by increasing the level of circulating PAF-AH by pretreating the mice with rPAF-AH. In both sets of experiments, the animals were protected from PAF-induced sudden death (Table 2). Furthermore, structurally related inactive lyso analogs of PAF were unable to mimic the effects of PAF, even when the concentrations of the lyso analogs were increased by 10-fold (Table 3). These experiments firmly establish the role of PAF-R in PAF-induced death.

**Table 1:** Strain-specific effects of PAF in murine species. Five-microgram aliquots of PAF were taken from a stock of 5 mg/mL in methanol, evaporated under a stream of nitrogen, reconstituted to a final volume of 0.5 mL in phosphate buffered saline containing 0.1% albumin, and sonicated. Then, the 5 μg doses of PAF were intraperitoneally injected into 3 strains of mice: Swiss albino, C57BL/6J, and BALB/c (n = 6 per strain). The mice were monitored for survival for up to 6 days. All of the Swiss albino mice that received PAF died within 15–20 min, whereas none of the C57BL/6J or BALB/c mice injected with PAF died. The results are representative of 3 individual trials.

Strain	Treatment	Death/total	%Survival
Swiss albino	Vehicle	0/6	100
	PAF (5 μg/mouse)	6/6	0
C57BL/6J	Vehicle	0/6	100
	PAF (5 μg/mouse)	0/6	100
BALB/c	Vehicle	0/6	100
	PAF (5 μg/mouse)	0/6	100

**Table 2:** PAF induces sudden death in Swiss albino mice. Swiss albino mice were divided into 9 groups containing 6 animals each. The indicated amounts of WEB-2086 or BN-52021 were aliquoted from a stock in DMSO, brought up to a total volume of 0.5 mL in PBS, and injected intraperitoneally into animals 30 min before PAF (5 μg/mouse) was injected. Animals that received rPAF-AH (25 μg/mouse) were intraperitoneally injected with it 30 min before being injected with PAF (5 μg/mouse), as were the animals that received the vehicle for rPAF-AH (sodium citrate, sucrose, pluronic, and Tween-80). All the animals injected with PAF alone (5 μg/mouse) died within 15–20 min. In contrast, all the animals that received either a PAF-R antagonist or rPAF-AH 30 min before being injected with a lethal dose of PAF survived. All animals were monitored for survival for up to 6 days. The results are representative of 3 individual trials.

Treatment	Death/total	%Survival
Vehicle	0/6	100
PAF (5 μg/mouse)	6/6	0
rPAF-AH (25 μg/mouse)	0/6	100
Vehicle for rPAF-AH	0/6	100
rPAF-AH (25 μg/mouse) + PAF (5 μg/mouse)	0/6	100
BN-52021 (20 mg/kg)	0/6	100
BN-52021 (20 mg/kg) + PAF (5 μg/mouse)	0/6	100
WEB-2086 (20 mg/kg)	0/6	100
WEB-2086 (20 mg/kg) + PAF (5 μg/mouse)	0/6	100

**Table 3:** Biologically inactive structural analogs of PAF are not lethal to Swiss albino mice. Aliquots of lysoPAF and lysoPC were taken from a stock of 5 mg/mL in methanol, dried under nitrogen, reconstituted in PBS containing 0.1% albumin, and sonicated. The indicated doses of each were brought up to a total volume of 0.5 mL with PBS containing 0.1% albumin and intraperitoneally injected into the mice. Animals injected with the vehicle (PBS containing 0.1% albumin) received the same volume. PAF (5 μg/mouse) was used as a positive control; all the animals injected with PAF died within 15–20 min. No mortality was observed for the animals intraperitoneally injected with the lyso analogs at doses equivalent to that used for PAF or 10-fold higher (50 μg/mouse). All the animals were monitored for survival for up to 6 days. The results are representative of 3 individual trials.

**Treatment**	**Death/total**	**% Survival**
Vehicle	0/6	100
PAF (5 μg/mouse)	6/6	0
LysoPAF (5 μg/mouse)	0/6	100
LysoPAF (50 μg/mouse)	0/6	100
LysoPC (5 μg/mouse)	0/6	100
LysoPC (50 μg/mouse)	0/6	100

### Hyperactivation of TLR-4 delays PAF-induced sudden death

**Fig 2:**
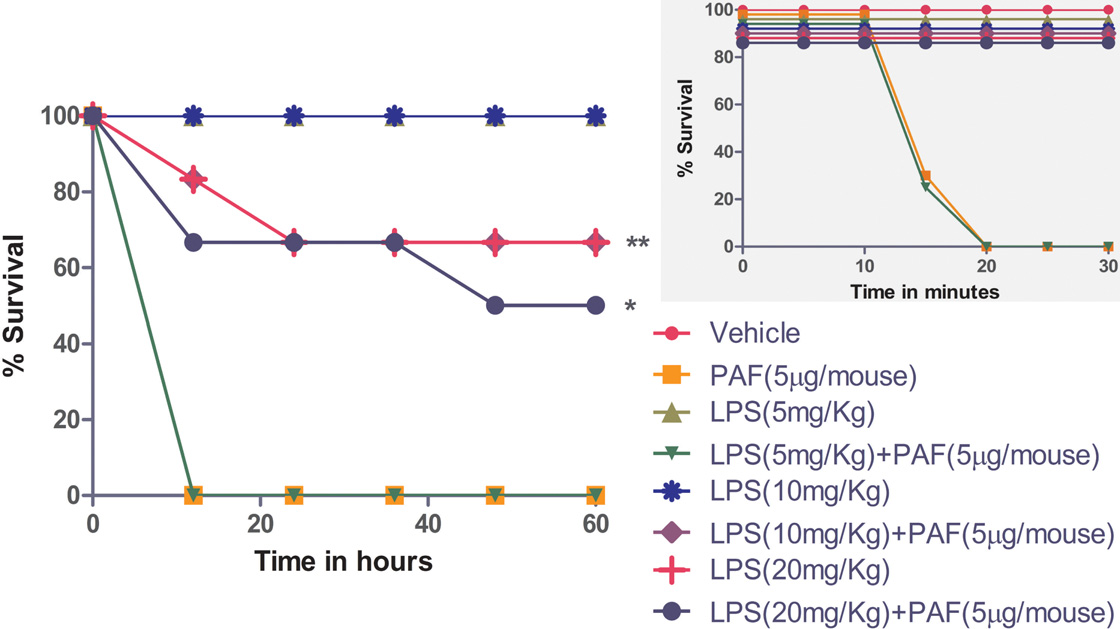
Hyperactivation of TLR-4 delays PAF-induced sudden death in Swiss albino mice. Animals were divided into 8 groups, each containing 6 animals. Three groups were intraperitoneally injected with LPS alone (5, 10, or 20 mg/kg body wt) dissolved in 0.5 mL PBS. Three other groups underwent intraperitoneal injections of LPS (5, 10, or 20 mg/kg body wt) immediately followed by PAF (5 μg/mouse) in 0.5 mL of PBS containing 0.1% albumin. No mortality was observed in the group injected with 10 mg/kg LPS, whereas 30% of the animals injected with 20 mg/kg LPS died in 5–24 h. All the animals injected with PAF alone (5 μg/mouse) and in conjunction with 5 mg/kg LPS died in 15–20 min, whereas a delay in PAF-induced death (2–48 h) was observed when higher doses of LPS (10 and 20 mg/kg) were used. Sixty-six percent of the animals that received PAF (5 μg/mouse) + 10 mg/kg LPS and 50% that received 20 mg/kg LPS + PAF (5 μg/mouse) survived for 6 days. The results are representative of 3 individual trials. *P<0.05 and **P<0.001, as compared with PAF-challenged mice. The *inset* graph represents survival rate of animals in minutes for the first 30 min after injection.

In the next series of experiments, PAF (5 μg/mouse) was injected into mice concomitantly with LPS (5 or 10 mg/kg body wt). LPS alone was not lethal at these doses, unless it was given in combination with D-galactosamine, a hepatotoxicant known to sensitize animals to TLR-4 agonists (data not shown) [73]. PAF alone (5 μg/mouse) caused sudden death, and injecting it simultaneously with a low dose of LPS (5 mg/kg body wt) did not alter this effect of PAF (Fig 2). However, using a higher dose of LPS (10 mg/kg body wt) delayed PAF-induced death to 2–48 h after injection, and 66% of the animals that received the higher LPS dose survived the full 6 days.

In the next experiments, we increased the LPS dose to 20 mg/kg body wt. Similar to the results with 10 mg/kg LPS, simultaneous injection of 20 mg/kg LPS and PAF (5 μg/mouse) delayed PAF-induced sudden death, and 50% of the animals that received this treatment survived for the full 6 days, presumably because of LPS cross-tolerance (Fig 2). However, 30% of the animals that received 20 mg/kg LPS alone died 5–24 h after injection (Fig 2). The amount of time PAF-induced death was delayed due to LPS cross-tolerance varied from animal to animal. LPS cross-tolerance was found to be time-dependent, with delay in PAF-induced death being seen when PAF was administered simultaneously or 30 min after the LPS injection but not when it was administered after longer periods (Table 4). Furthermore, we found that LPS cross-tolerance was not due to an abundant endogenously generated secondary mediator [74] because injecting naive mice with 100 μL of serum from mice injected with 20 mg/kg LPS + PAF (5 μg/mouse) 30 min before giving them a lethal dose of PAF (5 μg/mouse) did not delay PAF-induced death (Table 5). Also, injecting mice with a sublethal dose of LPS alone (50 μg) for 8 days did not protect them from a lethal dose of PAF (5 μg/mouse) given on day 9 (data not shown), suggesting the temporal nature of cross-tolerance.

**Table 4:** LPS cross-tolerance to PAF-induced lethality in Swiss albino mice is temporal. Mice were divided into 6 groups containing 6 animals per group. PAF (5 μg/mouse) was intraperitoneally injected together with LPS (20 mg/kg body wt) or at 30 min, 3 h, or 6 h after LPS was injected. All the animals injected with PAF alone (5 μg/mouse) or at 3 h or 6 h after receiving LPS (20 mg/kg) died in 15–20 min. In the group that received PAF concomitantly with LPS (20 mg/kg body wt), 33.4% of the animals died within 24 h, and 66.6% survived for 6 days. In the group that received PAF (5 μg/mouse) 30 min after LPS (20 mg/kg) was administered, the time until death was delayed to 12–24 h. All the animals were monitored for survival for up to 6 days. The results are representative of 3 individual trials.

**Treatment**	**Death/total**	**Death time**	**% Survival**
PAF (5 μg/mouse)	6/6	15–20 min	0
LPS (20 mg/kg)	2/6	40–48 h	66.6
LPS (20 mg/kg) + PAF (5 μg/mouse) (co-injection)	2/6	30 min-24 h	66.6
LPS (20 mg/kg) 30 min before PAF (5 μg/mouse)	6/6	12–24 h	0
LPS (20 mg/kg) 3 h before PAF (5 μg/mouse)	6/6	15–20 min	0
LPS (20 mg/kg) 6 h before PAF (5 μg/mouse)	6/6	15–20 min	0

**Table 5:** LPS cross-tolerance is not transferable. Mice were divided into 4 groups containing 4 animals each. In the initial phase of this experiment, animals were intraperitoneally injected with 0.5 mL of PBS or with LPS (20 mg/kg) + PAF (5 μg/mouse). These animals were then anesthetized, and their blood was collected 1 h after treatment. In the second phase of this experiment, 100 μL of the serum obtained from the mice treated with PBS or LPS (20 mg/kg body wt) + PAF (5 μg/mouse) was intraperitoneally injected into the other 2 groups of mice 30 min before they were injected with PAF (5 μg/mouse). Half of the mice in the group that originally received PAF (5 μg/mouse) + LPS (20 mg/kg) survived, but the serum from these animals failed to protect the naive animals injected with PAF (5 μg/mouse) 30 min after receiving the serum. The results are representative of 3 individual trials.

**Treatment**	**Death/total**	**% Survival**
PBS	0/4	100
LPS (20 mg/kg) + PAF (5 μg/mouse)	2/4	50
Serum (from mice treated with PBS and collected at 1 h) 30 min before PAF (5 μg/mouse)	4/4	0
Serum (from mice treated with LPS [20 mg/kg] + PAF [5 μg/mouse] and collected at 1 h) 30 min before PAF (5 μg/mouse)	4/4	0

### Involvement of COX pathway in PAF-induced sudden death and LPS cross-tolerance

PAF causes immediate mobilization of intracellular calcium with subsequent phospholipase A_2_-mediated release of arachidonic acid, which is then converted to bioactive eicosanoids by COXs [75]. Pretreating mice with either a 10 or 20 mg/kg body wt dose of aspirin (a non-specific COX inhibitor) 30 min before injecting them with a lethal dose of PAF prevented death in 66.6% and 71.4% of the animals, respectively (Table 6). Administering aspirin (20 mg/kg body wt) 30 min before simultaneously injecting PAF (5 μg/mouse) and a high dose of LPS (20 mg/kg) conferred complete protection against delayed death (Table 6). Interestingly, no mortality was observed when animals were pretreated with NS-398 (20 mg/kg body wt) 30 min before being injected with PAF (5 μg/mouse), LPS (20 mg/kg), or the combination of LPS (20 mg/kg) + PAF (5 μg/mouse) (Table 7), suggesting that the COX pathway may be involved in PAF-mediated death and the cross-tolerance exerted by hyperactivated TLR-4 in Swiss albino mice.

**Table 6:** Aspirin partially protects Swiss albino mice from PAF-induced lethality and amplifies the cross-tolerance exerted by hyperactivated TLR-4. Mice were divided into 11 groups. Three groups were intraperitoneally injected with a 10 mg/kg dose of aspirin 30 min before being injected with the indicated doses of PAF, LPS, or both. Three other groups were intraperitoneally injected with a 20 mg/kg dose of aspirin 30 min before receiving PAF, LPS, or both. The 20 mg/kg dose of aspirin partially protected the animals from PAF-induced sudden death, whereas it completely protected the animals from delayed death due to PAF (5 μg/mouse) plus a high dose of LPS (20 mg/kg). The animals were monitored for survival for up to 6 days. The results are representative of 3 individual trials.

**Treatment**	**Death/total**	**% Survival**
Vehicle	0/6	100
PAF (5 μg/mouse)	6/6	0
LPS (20 mg/kg)	2/6	66.6
LPS (20 mg/kg) + PAF (5 μg/mouse)	2/6	66.6
Aspirin (10 mg/kg)	0/6	100
Aspirin (20 mg/kg)	0/6	100
Aspirin (10 mg/kg) 30 min before PAF (5 μg/mouse)	2/6	66.6
Aspirin (20 mg/kg) 30 min before PAF (5 μg/mouse)	2/7	71.4**
Aspirin (10 mg/kg) 30 min before LPS (20 mg/kg)	2/6	66.6
Aspirin (20 mg/kg) 30 min before LPS (20 mg/kg)	0/6	100^#^
Aspirin (10 mg/kg) 30 min before LPS (20 mg/kg) + PAF (5 μg/mouse)	3/6	50
Aspirin (20 mg/kg) 30 min before LPS (20 mg/kg) + PAF (5 μg/mouse)	0/7	100^●^

**P<0.01 as compared with PAF-challenged mice

^#^*P*<0.01 as compared with LPS-challenged mice, and

^●^*P*<0.05 as compared with LPS + PAF-challenged mice.

**Table 7:** Specific COX-2 inhibitor confers complete protection against both PAF-induced lethality and delayed death due to PAF and hyperactivated TLR-4. Mice were divided into 7 groups containing 6 animals each. Three groups received an intraperitoneal injection of NS-398 (20 mg/kg body wt) 30 min before receiving PAF, LPS, or a combination of both. NS-398 (20 mg/kg body wt) completely abolished PAF-induced sudden death and enhanced the cross-tolerance effect exerted by the high dose of LPS (20 mg/kg). The animals were monitored for survival for up to 6 days. The results are representative of 3 individual trials.

Treatment	Death/total	% Survival
Vehicle	0/6	100
PAF (5 μg/mouse)	6/6	0
LPS (20 mg/kg)	3/6	50
LPS (20 mg/kg) + PAF (5 μg/mouse)	2/6	66.6
NS-398 (20 mg/kg)	0/6	100
NS-398 (20 mg/kg) 30 min before PAF (5 μg/mouse)	0/6	100
NS-398 (20 mg/kg) 30 min before LPS (20 mg/kg)	0/6	100
NS-398 (20 mg/kg) 30 min before LPS (20 mg/kg) + PAF (5 μg/mouse)	0/6	100

### Histological assessment of lungs and liver

**Fig 3:**
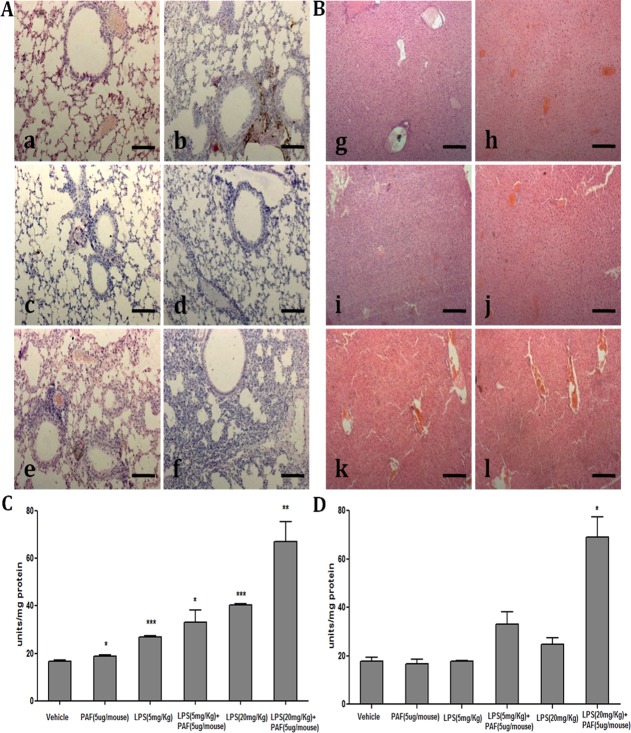
Architecture of lung and liver sections stained with hematoxylin and eosin and myeloperoxidase levels in each organ. **(A)** Lung sections from animals injected with **a.** vehicle, **b.** PAF (5 μg/mouse), **c.** 5 mg/kg LPS, **d.** 20 mg/kg LPS, **e.** 5 mg/kg LPS + PAF (5 μg/mouse), or **f.** 20 mg/kg LPS + PAF (5ug/mouse). Magnification = 20X; and scale bar represents 200 μm **(B)** Liver sections from animals injected with **g.** vehicle, **h.** PAF (5 μg/mouse), **i.** 5 mg/kg LPS, **j.** 20 mg/kg LPS, **k.** 5 mg/kg LPS + PAF (5ug/mouse), or **l.** 20 mg/kg LPS + PAF (5 μg/mouse). Magnification = 10X; and scale bar represents 100 μm. The images are representative sections from each group. Lung sections from mice injected with LPS (20 mg/kg) + PAF showed significant thickening of the alveolar septum, vascular congestion, and cytoplasmic vacuolization of the airways, whereas liver sections showed necrosis and distorted architecture. (**C**, **D**) The extent of polymorphonuclear leukocyte (PMN) infiltration into the organs was indirectly estimated by quantifying the level of the PMN-specific enzyme MPO in tissue homogenates of lung (C) and liver (D) from the respective groups. The results are presented as units/mg of protein ± SD. *P<0.05; **P<0.01; ***P<0.001 as compared with mice injected with vehicle. Although hyperactivation of TLR-4 via LPS conferred protection against PAF-induced sudden death, it did not prevent inflammation, as evident from the histological analysis.

The lungs and liver are the major organs affected by endotoxemia [76–79], and the extent of leukocyte infiltration and architecture distortion in these organs correlates with the severity of the damage [80–81]. Lung and liver tissue from the mice that received LPS alone or in combination with PAF showed substantial leukocyte infiltration and altered structural integrity, as seen in hematoxylin and eosin-stained sections. Such pulmonary sequestration and activation of leukocytes can be the direct effect of LPS, as shown by Haslett et al [80] in their rabbit model. Lung sections showed significant thickening of the alveolar septum, vascular congestion, and cytoplasmic vacuolization of the airways (Fig 3). In the animals that received PAF alone (5 μg/mouse), we did not see much sequestration of leukocytes, probably because samples were taken at an early time point due to the fact that the animals died within 15–20 min (Fig 3). Although LPS (20 mg/kg body wt) delayed PAF-induced death, histological assessment of tissues from these animals showed that the lungs had thickened alveolar septae with massive leukocyte infiltration and that the liver had marked necrosis and highly distorted architecture, as compared to the normal lobular architecture seen in the vehicle-treated group (Fig 3). These findings indicate that there was no reduction in inflammation during LPS cross-tolerance.

### Myeloperoxidase (MPO) activity in lungs and liver

To assess the extent of inflammation more quantitatively, we determined the level of MPO, which is mainly derived from infiltrating leukocytes, in tissue homogenates from the experimental animals. The lungs and liver of animals injected with LPS alone (5 or 20 mg/kg body wt) showed an increase in MPO levels, as compared to that in vehicle-and PAF-injected (5 μg/mouse) animals (Fig 3). A substantial increase in MPO activity was observed in the lungs and liver of animals treated with LPS (20 mg/kg) + PAF (5 μg/mouse) (Fig 3), which supports the histological observations of no reduction in inflammation during LPS cross-tolerance.

### LPS cross-tolerance does not reduce the level of circulating TNF-α or increase the level of IL-10

**Fig 4:**
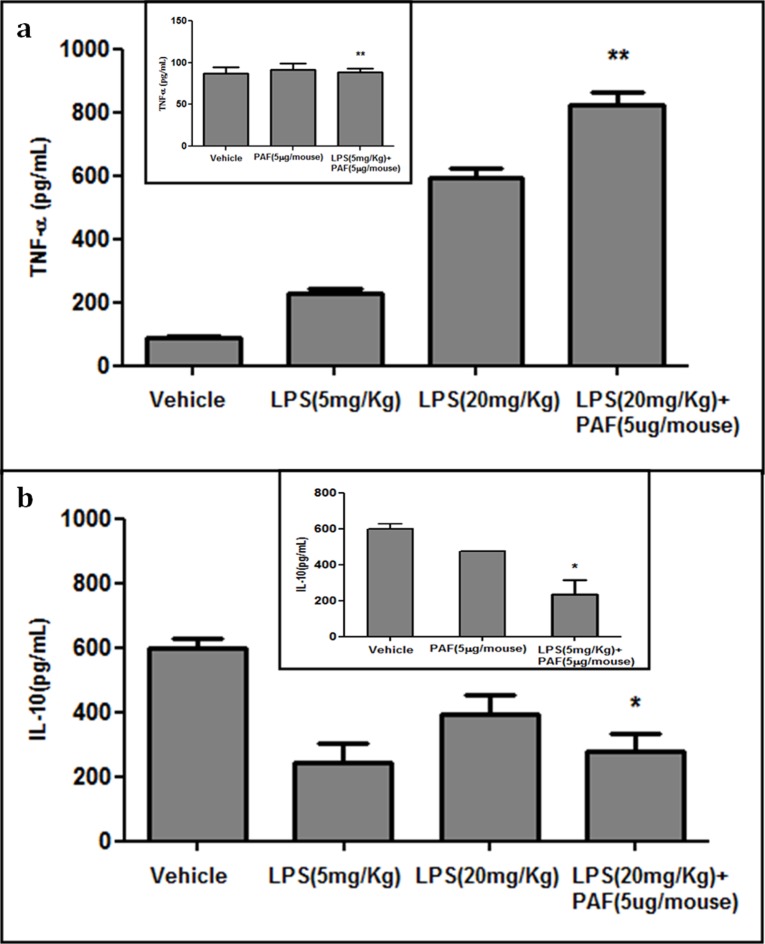
Serum levels of TNF-α and IL-10 in mice challenged with PAF in the presence and absence of LPS. (**a**, **b**) Blood was collected from the animals injected with PAF alone (5 μg/mouse) or with 5 mg/kg LPS + PAF (5 μg/mouse) 7 min after challenge, whereas blood was collected 4 h after challenge for the other animals. Serum was then separated from the blood for analysis. The levels of TNF-α (a) and IL-10 (b) were measured using ELISA kits, as per the manufacturer’s instructions. The insets show the TNF-α and IL-10 levels for the mice injected with vehicle or PAF (5 μg/mouse) or 5 mg/kg LPS + PAF. All values are expressed as pg/mL and presented as mean ± SD (n = 3). *P<0.05; **P<0.01, as compared with mice injected with vehicle. LPS cross-tolerance to PAF-induced sudden death neither increased the level of circulating anti-inflammatory IL-10 nor decreased the level of pro-inflammatory TNF-α.

The levels of TNF-α, a key cytokine, are known to be elevated during many inflammatory conditions, including experimental sepsis [82–83]. In contrast, soluble factors, including anti-inflammatory cytokine IL-10, have been shown to contribute to the suppression of inflammation during cross-tolerance [84–85]. Therefore, we measured the serum levels of TNF-α and IL-10 in mice injected with LPS and PAF. Intraperitoneal injection of LPS alone (5 or 20 mg/kg body wt) significantly increased serum TNF-α levels (Fig 4A). Because animals injected with PAF alone (5 μg/mouse) or with LPS (5 mg/kg) + PAF (5 μg/mouse) died within 15–20 min, blood could only be collected from these animals within this short time and did not show any rise in TNF-α levels (Fig 4A). Interestingly, although LPS (20 mg/kg body wt) delayed PAF-induced death, it failed to reduce circulating TNF-α levels, suggesting again that inflammation is not diminished during cross-tolerance (Fig 4A). For unknown reasons, we failed to observe an increase in anti-inflammatory IL-10 during cross-tolerance (Fig 4B).

## Discussion

LPS is the most abundant and widely used bacterial endotoxin that activates the pattern-recognizing TLR-4 receptor, the downstream signaling events of which are critical in the pathogenesis of many inflammatory diseases, including sepsis [4–5]. PAF, one of the most potent pro-inflammatory mediators of the innate immune system, exerts its effects via the PAF-R and is believed to be partly responsible for the complications of sepsis [80, 86–90]. Given that these ligands activate the innate immune system through different receptors, one would expect that their combined actions would exacerbate inflammatory responses. Moreover, previous studies have shown that endotoxins are good inducers of PAF biosynthesis in human neutrophils [9, 91], and results from overexpressing or deleting PAF-R in animal models suggest that PAF plays a role in endotoxemia [38,40].

Although the vast majority of studies in the literature have suggested that both PAF and PAF-like lipids are pro-inflammatory, a few studies have shown that PAF and PAF-like lipids may play a protective role in endotoxemia [50–53]. However, we found that PAF is lethal to Swiss albino mice at a dose of 5 μg/mouse. One possible reason for the discrepancy between our results and those of others could be differences in the mouse strain used. For example, in reports by Bochkov et al [50] and Jeong et al [52], the protective effects of PAF and PAF-like lipids were found using C57BL/6J and BALB/c mice, respectively, whereas we used Swiss albino mice—a strain that is exquisitely sensitive to PAF. When we assessed the strain-specific effects of PAF in murine species, we found that intraperitoneal administration of 5 μg/mouse was lethal to Swiss albino mice but not to C57BL/6J and BALB/c mice (Table 1). We provide 4 lines of evidence that PAF-induced sudden death in Swiss albino mice was directly due to PAF: (1) the effect of PAF was dose-dependent (Fig 1), (2) two structurally different but specific PAF-R antagonists blocked the lethal effects of PAF (Table 2), (3) increasing the circulating levels of PAF-AH, which hydrolyses PAF and PAF-like lipids, also protected mice from PAF-induced death (Table 2), and (4) inactive lyso derivatives of PAF were unable to replicate the effects of PAF, even at a concentration 10 times higher than that required for PAF (Table 3), suggesting that lysoPAF/lysoPC are not rapidly acetylated by PAF synthase to make lethal amounts of PAF [91–92] that could then cause its effects. We also noticed that a single rPAF-AH treatment (25 μg/mouse) could protect the animals from PAF-induced death for about a week, even when a lethal dose of PAF (5 μg/mouse) was given every day (data not shown). This week-long protection was probably not due to the presence of circulating rPAF-AH but may have been due to endogenously generated PAF-AH in response to PAF injection, as shown by Wang et al [93]. Henderson et al [48] have reported that exogenously administered rPAF-AH stays in circulation for 6–8 h in BALB/c mice, whereas Gomes et al [86] have shown that the half-life of rPAF-AH can be extended to 24 h in Swiss albino mice.

Simultaneously administering a low dose of LPS (5 mg/kg body wt) with PAF did not modulate the lethal effects of PAF (Fig 2), but higher doses of LPS (10 and 20 mg/kg body wt) delayed PAF-induced sudden death, probably because of endotoxin cross-tolerance (Fig 2). Although, higher doses of LPS temporally delayed the detrimental consequences of PAF, this cross-tolerance could not be attributed to either a decrease in circulating TNF-α levels (Fig 4A) or an increase in IL-10 levels (Fig 4B), as suggested in a few studies [59, 61, 74], nor could it be attributed to a reduction in leukocyte infiltration into the lungs and liver (Fig 3), as evident from our histological examination. Similarly, the levels of the leukocyte marker enzyme MPO did not decrease in the lungs (Fig 3C) and liver (Fig 3D), further supporting the idea that inflammation was not diminished during cross-tolerance.

LPS cross-tolerance is the hyporesponsiveness of animals/isolated cells to an immunostimulatory challenge after exposure to a sublethal dose of LPS. A few studies have attributed this hyporesponsiveness to endogenously generated anti-inflammatory mediators, such as IL-10, TGF-β [84–85], altered G-protein content, phospholipase D, IkB kinase, GSK-3, SOCS-1, IRAK-1, or SHIP [54, 60]. However, we tested 2 of the candidate mediators, TNF-α and IL-10, and failed to see an increase in the level of circulating anti-inflammatory IL-10 (Fig 4B) or a decrease in the level of TNF-α (Fig 4A) during cross-tolerance. We also found that cross-tolerance was temporal (Table 4) and not transferable (Table 5).

To better understand the phenomenon of cross-tolerance, we evaluated the effects of 2 COX inhibitors: aspirin and NS-398. Aspirin is traditionally used as a non-steroidal anti-inflammatory drug [64], but may have other effects as well [94]. In our study, aspirin (20 mg/kg) partially protected mice (71.4%) from PAF-induced sudden death, whereas it completely protected the animals from the delayed death caused by LPS (20 mg/kg) + PAF (Table 6). Aspirin is known to inhibit the activation of NF-kB [94], along with COX, and converts COX-2 to acetylated COX-2, which may then generate 17(R)-Resolvin D1 via its peroxidase activity [95–96]. Although we did not measure the levels of resolvins in our study, the aspirin may have formed resolvins, which are endogenous anti-inflammatory mediators generated to resolve inflammation. To test the possible involvement of COX-2, we used a specific COX-2 inhibitor (NS-398) that is less likely to generate aspirin-triggered resolvins. When tested at a dose equivalent to that used for aspirin, NS-398 completely protected the animals from the lethal effects of PAF and also alleviated the cross-tolerance effects of the higher dose of LPS (Table 7). The blunting effects of COX inhibitors seen in this study suggest that COX-driven eicosanoids may be involved in PAF-induced lethality and cross-tolerance induced by LPS. This may have far-reaching implications for our understanding of the previously unrecognized role of the COX pathway in endotoxin cross-tolerance and may provide new avenues for therapeutic interventions to prevent infectious disease complications, such as sepsis. Currently, we are trying to understand the fine details of cross-tolerance and the involvement of other toll receptors in cross-tolerance in this murine model of endotoxemia.
